# A Novel Surface Acoustic Wave Sensor Array Based on Wireless Communication Network

**DOI:** 10.3390/s18092977

**Published:** 2018-09-06

**Authors:** Yong Pan, Ning Mu, Bo Liu, Bingqing Cao, Wen Wang, Liu Yang

**Affiliations:** 1 State Key Laboratory of NBC Protection for Civilian, Beijing 102205, China; panyong71@sina.com.cn (Y.P.); sdmuta@163.com (N.M.); s13810206336@163.com (B.L.); 13126584199@163.com (B.C.); 2 State Key Laboratory of Acoustics, Institute of Acoustics, Chinese Academy of Sciences, Beijing 100190, China

**Keywords:** SAW sensor array, wireless communication, network, detection, gases

## Abstract

A novel surface acoustic wave (SAW) sensor array based on wireless communication network is prepared. The array is composed of four SAW sensors, a wireless communication network module, and a global positioning system (GPS) module. The four SAW sensors of the array are coated with triethanolamine, polyepichlorohydrin, fluoroalcoholpolysiloxane, and L-glutamic acid hydrochloride to detect hydrogen sulfide (H_2_S), 2-chloroethyl ethyl sulfide (CEES), dimethylmethylphosphonate (DMMP), and ammonia (NH_3_) at film thicknesses of 50–100 nm. The wireless communication network module consists of an acquisition unit, a wireless control unit, and a microcontroller unit. By means of Zigbee and Lora technologies, the module receives and transmits the collected data to a PC work station in real-time; moreover, the module can control the sensor array’s working mode and monitor the working status. Simultaneously, the testing location is determined by the GPS module integrated into the SAW sensor array. H_2_S, CEES, DMMP, and NH_3_ are detected in 300 m at different concentrations. Given the practical future application in environment in the future, the low, safe concentrations of 1.08, 0.59, 0.10, and 5.02 ppm for H_2_S, CEES, DMMP, and NH_3_, respectively, are detected at the lowest concentration, and the sensitivities of different sensors of the sensor array are 32.4, 14.9, 78.1 and 22.6 Hz/ppm, respectively. With the obtained fingerprints and pattern recognition technology, the detected gases can be recognized.

## 1. Introduction

Environment and public security are becoming increasingly important to human health and safety. With the development of the world, harmful gases or numerous other volatile organic compounds (VOCs), which are widely used in the industries, pose potential risks for serious diseases and environmental and explosion dangers. For example, hydrogen sulfide (H_2_S), a harmful gas, is a colorless toxic gas that can cause adverse effects on the human body at a low concentration of 2 ppm, and can be fatal with prolonged exposure at levels of 100 ppm [1]. Ammonia (NH_3_) is another harmful gas that is primarily released from combustion in chemical industries, and exposure to high ammonia concentration can result in life-threatening situations [2]. By contrast, chemical warfare agents (CWAs) are powerful weapons and a threat to civil safety because of their extremely high hazard and potential lethality. Particularly in the recent years, numerous terrorism events involving CWAs that resulted in numerous casualties or injuries have been reported. Therefore, the threat of CWAs is crucial to national security and world affairs. Fast and sensitive detection of industrial harmful gases, VOCs, and CWAs is highly essential in protecting humans [3,4,5]. In the last 20 years, sensor technology has received increasing attention for ubiquitous sensing. As important sensing technologies, surface acoustic wave (SAW) devices have exhibited promising characteristics because of their high sensitivity, fast response, excellent specificity, reversibility, battery-powered operation, small size, and low cost, thus offering extensive potential use in the future. Several reviews presented the mechanism for chemical specificity and chemically specific layers of SAW sensors; numerous studies focused on the detection of industrial harmful gases, VOCs, or CWAs [6,7,8].

A SAW sensor array is typically required to identify the detected gases accurately, and each sensor in the array should be coated with different sensing films. By simultaneous data gathering and analysis of substantial data and pattern recognition methods, the targets can be discriminated; thus, the chemical sensor array can play an important role in the analysis of gas analytes [9]. CWAs have been successfully detected by different SAW arrays [7,10], and several harmful gases, such as H_2_S, NH_3_, and NO_2_, are also detected by SAW technology [1,2,11,12,13]. However, most currently available SAW sensor arrays for detecting industrial harmful gases, VOCs or CWAs are manually operated, extremely costly, and time consuming [14]; thus, real-time, continuous, long-term monitoring sensors are attractive for in situ detection, possibly reducing the need for manual operation and expensive off-site analyses.

Passive wireless SAW resonant sensors can meet the abovementioned requirement because of their application to a harsh environment and simple installation; wiring and penetration into the harsh environment is not needed [15,16]. In recent years, several SAW embodiments have been proposed for wireless, passive SAW sensors [17,18], and these may be practical for portable or wireless sensor networks (WSN). Numerous designers presented the option of integrating general WSN platforms with sensor devices for a variety of applications [19,20,21], and several wireless and passive SAW-based sensors have been used to detect organophosphorus compounds [22], CO_2_, NO_2_ or humidity [23,24,25]. Several studies on wireless chemical sensors and algorithms for SAW sensors were reviewed [26,27]. Meanwhile, certain design technologies, such as coupling of modes, which is an efficient technique for SAW devices, are used before fabrication to find optimal design parameters of the SAW sensors [28]. All of the abovementioned works provide a good starting point for the development of real practical wireless SAW chemical sensors.

In the last decade, with the development of computer systems, particularly the evolution of processor systems and advances in communication networks, WSN is becoming the focus of sensor studies and is regarded as one of the most promising future technologies [29,30,31]. An increasingly large market is trying to apply WSN to sensor technology, and sensors combined with a wireless communication network are becoming the current trend. Meanwhile, global positioning systems (GPSs) have been used in WSN, enabling the establishment of a wireless sensors communication network to monitor target gases. As the prominent technologies, SAW sensor arrays are tested for use in this monitoring platform [18,21]. In this study, we present a new SAW sensor array based on WSN and established a novel wireless communication platform, which successfully detected harmful gases (H_2_S, NH_3_) and warfare agent simulants (DMMP, CEES, simulants of sarin and mustard gas) within 300 m and located the testing position. Although wireless communication is a big challenge in the study of sensors, the WSN that we suggest here may be used in environmental protection or in any other future monitoring application.

## 2. Materials and Methods

### 2.1. Reagents and Instruments

**Figure 1:**
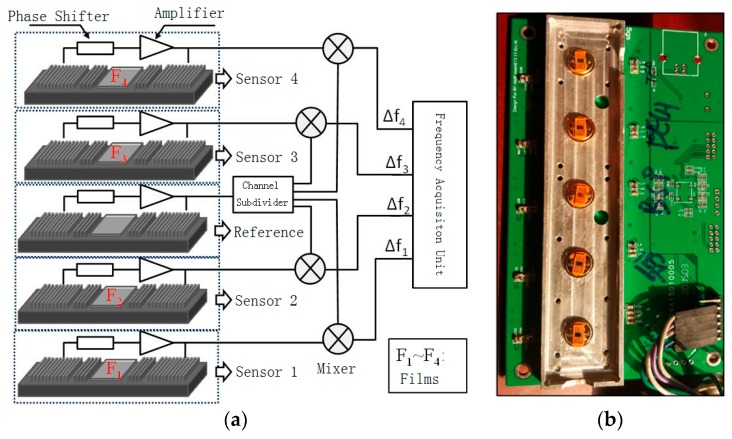
(**a**) Schematic of the SAW sensor array (sensor 1, coated with TEA; sensor 2, coated with PECH, sensor 3, coated with SXFA, sensor 4, coated with GAH); (**b**) The photo of the SAW sensor array.

The SAW sensor array used in this work is fabricated by Institute of Acoustic of Chinese Academy of Science. The array consisted of a five-channel sensing device with two-port resonator configuration: one device is naked as the reference to compensate the influence of temperature and pressure, and the other four devices are coated with different films for sensing target gases. Each device is fabricated lithographically on ST-X quartz, the corresponding working frequency is set to 300 MHz. The proposed SAW resonator is composed of two interdigital transducers (IDTs) and two reflector located in each side of the IDT. Also, the Al-electrodes are used to build the SAW devices. To deposit sensitive film easily, the resonant cavity of 2 mm^2^ between the IDTs was designed. The developed sensing devices are collected into the differential oscillation loop, which is composed of the phase shifter, amplifier, and mixer described in Figure 1. The mixed oscillation frequency signal is collected to evaluate the gases to be detected.

Triethanolamine (TEA), polyepichlorohydrin (PECH), and L-glutamic acid hydrochloride (GAH) were purchased from Aladdin Chemical Reagent Company (Shanghai, China). Fluoroalcoholpolysiloxane (SXFA) was synthesized by our laboratory. The detected gases, H_2_S, and NH_3_ were provided by Beijing Haipu Company (Beijing, China), and DMMP and CEES were generated in our laboratory.

IP-LINK1223-5152 is an embedded wireless module based on IEEE 802.15.4/ZigBee technology launched by Helicomm Company (San Diego, CA, USA). E32 series wireless communication module is a wireless serial module that uses the LoRa spread spectrum technology made by Semtech Company (Camarillo, CA, USA) and works in the 410–441 MHz band (the default 433 MHz). RXM-GPS-FM-T is a Linx Technologies (London, UK) FM series GPS module. The accuracy of the horizontal position is 3 m, which supports a maximum of 66 channels and reaches a sensitivity value of −161 dBm.

### 2.2. Preparation of SAW Sensor Array

As mentioned above, H_2_S, CEES, DMMP, and NH_3_ were detected by SAW sensors coated with TEA, PECH, SXFA and GAH. All the films selected in this study are viscoelastic. TEA is a kind of alkaline film, atom S contained in H_2_S could combine with atom H contained in TEA by weak hydrogen-bond and van der Waals force, CEES could permeate into PECH easily for its good solubility, while the use of SXFA could form conjugated structure with P=O of organophosphorous compounds due to the –CF_3_ in SXFA, for GAH, NH_3_ could dissolve easily in GAH because of the rule of similarity. The sensing mechanisms of adsorption between detected gases and films had been discussed before, and the studies were effective. On these bases, TEA, PECH, SXFA and GAH were selected in this study as sensitive membrane materials to detect H_2_S, CEES, DMMP, and NH_3_, respectively.

The structure and photo image of the SAW sensor array used in this study are shown in Figure 1. Five channels are included in the sensor array: the uncoated middle channel is selected as the reference to delete the influence of temperature and pressure. The other four sensors (sensor 1, sensor 2, sensor 3, and sensor 4), which are coated with TEA, PECH, SXFA, and GAH, are used to detect H_2_S, CEES, DMMP, and NH_3_.

In preparing of SAW sensor array, all the films were prepared by dipping method, the operation method is as follows. First, TEA and GAH were prepared in ethanol solvent at a concentration of 0.2 mg/mL. PECH and SXFA were prepared using chloroform and acetone, respectively, at the concentration of 0.1 mg/mL. Second, to get the needed thickness of the films, by removing some volume solution having been prepared, after dipping on the surface of the SAW sensor and calculating, the needed thickness film could be gotten. For example, 1 μL of TEA solution was withdrawn and dipped on the coating area, which was dried with N_2_ at room temperature. TEA was deposited on the surface of the SAW device, and the thickness of TEA film was calculated using the formula below:

*m*_1_ = *ρv*_1_ = *ρsh* (1)

*m*_2_ = *cv*_2_ (2)

*h* = *cv*_2_/*ρs* (3)

In Formula (1), *m*_1_ (mg) is the amount of TEA on coating area, *ρ* (g/cm^3^) is the density of TEA, *v*_1_ (cm^3^) is the volume of TEA on the coating surface, *s* (cm^2^) is coating area, and *h* (cm) is the thickness of film. In Formula (2), *m*_2_ (mg) is the amount of TEA, *c* is the concentration of TEA solution prepared, and *v*_2_ is the volume removed (1 μL). *m*_1_ is equal to *m*_2_; therefore, the average thickness of TEA can be estimated using Formula (3) and is approximately 70 nm. The other three kinds of films are also prepared similarly.

### 2.3. Structure of the Wireless Communication SAW Sensor Array

**Figure 2:**
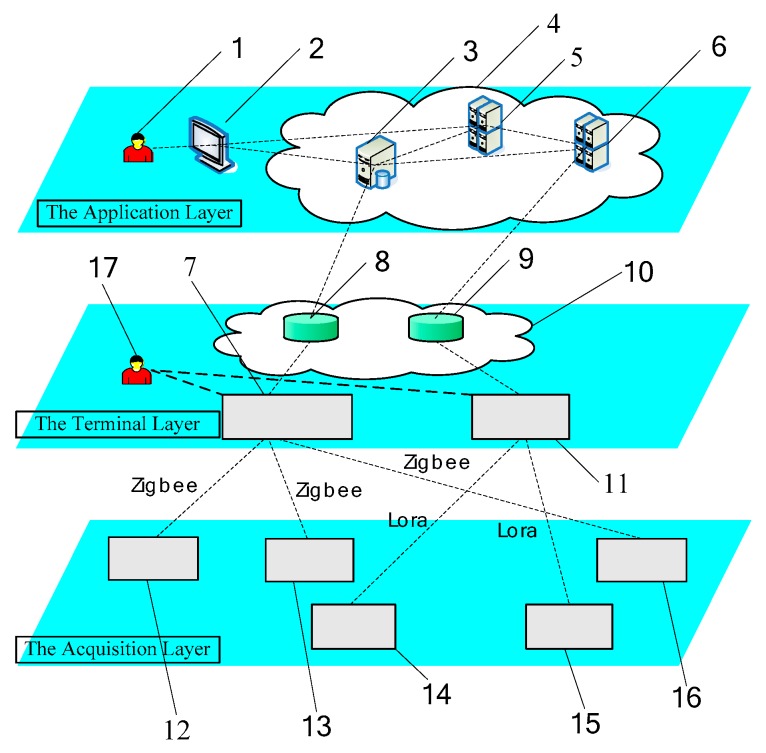
Detecting and wireless communication system of the SAW sensor array. 1—Decision Making User; 2—Display Platform; 3—Database Server; 4—Cloud Computing Platform; 5—Application Server 1; 6—Application Server 2; 7—Control Terminal 1; 8—Database 1; 9—Database 2; 10—Cable/Wireless Network; 11—Control Terminal 2; 12—SAW Sensor 1; 13—SAW Sensor 2; 14—SAW Sensor 3; 15—SAW Sensor 5; 16—SAW Sensor 4; 17—Operating User.

The wireless communication network module of the SAW sensor array system was designed and developed by our laboratory. This system consists of an acquisition layer, a terminal layer, and an application layer. The three parts are equipped together, forming the total system. A block diagram of the detection and wireless communication system of the SAW sensor array is shown in Figure 2.

The acquisition layer is located at the front of the whole system. The layer is composed of SAW sensor array, data acquisition module, GPS module, and Zigbee and Lora transceiver modules. As a wireless transceiver module, it not only collects the required data and position information but also transmits data to the terminal layer. The terminal layer, which includes the control terminal, database, and the wireless transceiver module, is called the client module. Its function is to connect the system by wired/wireless networks, provide access to the collection of SAW sensor data, control each sensor’s working mode, and analyze and detect/monitor the target gas types and information on concentration in the environment. The human computer interaction interface is divided into two functions, namely, instrument control operation and target gas information display.

The application layer is a server module composed of the cloud computing platform, data server, application server, and display platform. The layer is commonly used to receive information from the control terminal and provide information to the decision-making user to determine target gas information in the detection/monitoring environment. Meanwhile, the database server stores useful data, and the application and data-processing servers analyze the statistical data.

The total working procedure is operated as follows. When the target gas is exposed to the SAW sensor array in the acquisition layer, the detecting array data of frequency shifts are outputted and collected by the data acquisition module. Afterward, the data are transferred to the main control center by GPS module, Zigbee, and Lora transceiver modules. After processing of the original data by the main control center, the detecting array curves are displayed on computer screen in real-time.

### 2.4. Experiments Methods

The developed wireless SAW sensor array is evaluated at temperature/humidity of 19 °C and 20% RH. The sensor array is sealed in a gas chamber, and the detected gas enters the gas chamber and is exposed to the sensor arrays alternately via the gas path. Meanwhile, the personal computer used to process detection data and locate the detecting position is 300 m away. As mentioned above, the proposed SAW sensor system is composed of the SAW sensor array, the frequency signal acquisition module (FSAM), GPS module, Zigbee and Lora transceiver modules (wireless module). Hence, the sensor signal is collected by the FSAM, and trasmitted by the wireless module to the commercial received module in the PC, and then recorded and plotted by the PC using the homemade software.

## 3. Results

### 3.1. Electrical Characterization of the SAW Device

**Figure 3:**
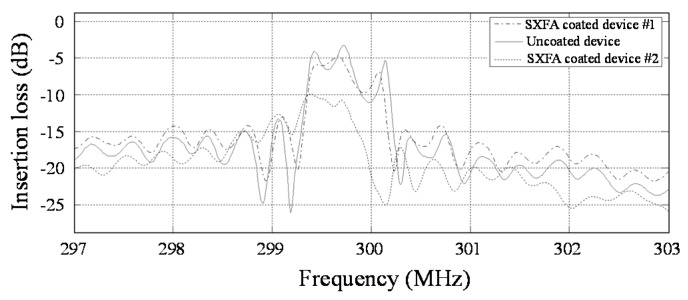
Measured frequency response of the prepared SAW sensing device before and after SXFA deposition (Device #1—50 nm, Device #2—200 nm).

The SAW sensing devices are electrically characterized by a network analyzer (Figure 3). Figure 3 indicates the frequency response of the prepared SAW sensing device before and after SXFA (50 and 200 nm) deposition. SXFA deposition not only induces frequency shift due to the mass loading but also evident acoustic attenuation because of the viscoelastic nature; moreover, a thick SXFA film produces large attenuation, which considerably increases the baseline noise of the sensor system. Thus, a thick polymer sensitive film should be avoided.

### 3.2. Stability Test and Gas Sensor Array Experiment

**Figure 4:**
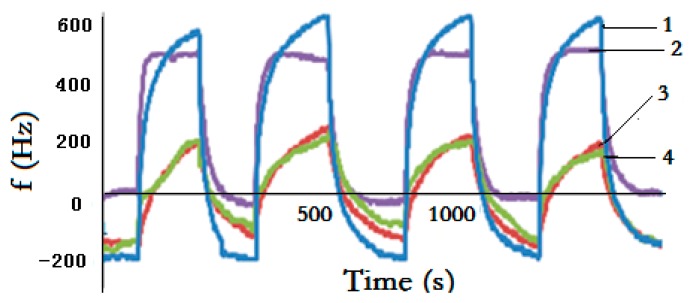
Four times responses and repeatability of sensor array to H_2_S. (1—sensor 1, 2—sensor 2, 3—sensor 3, 4—sensor 4).

Replicate experiments were conducted to validate the stability of the SAW sensor array. Under room conditions, H_2_S (7.2 ppm) with carrier gas N_2_ was selected for detection and passed through the SAW sensor array sequentially for four replicate experiments (Figure 4).

Notably, Figure 4 indicates a typical response profile obtained from four times consecutive testing, and the sensor array shows good reproducible run. Given its TEA coating and sensitivity to H_2_S, sensor 1 yielded frequency data that show a rapid increase upon exposure to H_2_S in the initial several seconds and the largest frequency shift upon reaching the adsorption equilibrium. By comparison, the frequency shifts of the other three sensors are lower; when H_2_S is removed by N_2_ injection, the sensor array returns to its initial baseline. The same phenomenon is expected to occur when CEES, DMMP, and NH_3_ are detected by the sensor array. These promising results exhibit that the sensor array offers fast response and excellent repeatability in detecting target gases.

**Figure 5:**
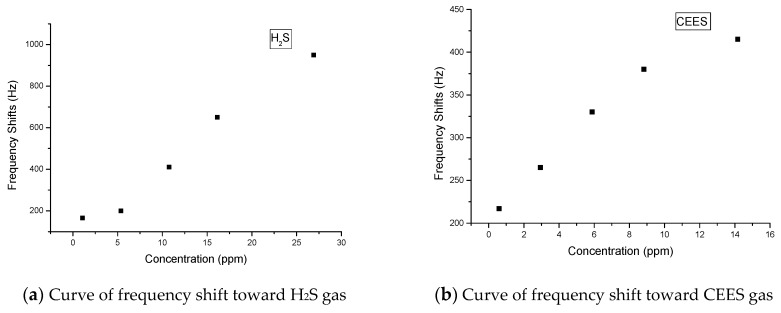

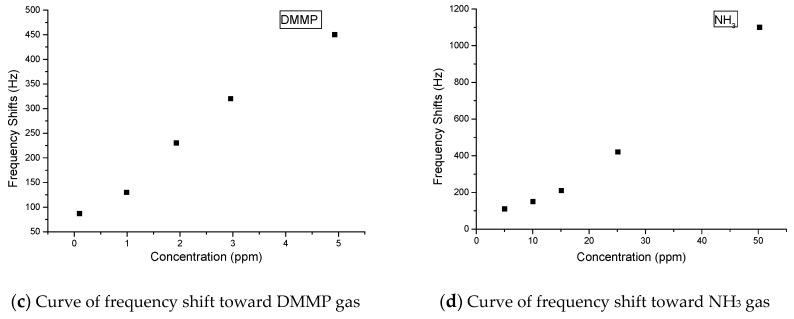
Response of the SAW sensor array to different concentration gases.

After the above experiment, different concentrations of H_2_S, CEES, DMMP, and NH_3_ are detected with the fabricated sensor array. Before experiments, the sensor array tends to stabilize after 20 min, frequency stability can reach 30 Hz h^−1^ in the stable status, and the results of the detection of the four gases are shown in Figure 5. As can be concluded using the figure, the responses for the four gases are sensitive at low concentrations; moreover, the sensor array can provide the signals of 165, 217, 87, and 110 Hz for H_2_S, CEES, DMMP, and NH_3_ at the concentrations of 1.08, 0.59, 0.1, and 5.02 ppm, respectively. These environmental concentrations are considered safe for humans. On the basis of the results, we suggest that the sensor array is promising for use in gas detection.

### 3.3. Establishment of Sensor Data Transmission Model

Reliable transmission models are essential to ensure the correctness and effectiveness of wireless data. Based on Zigbee technology, a WSN should consist of a base station and abundant nodes. The microprocessor processes the raw data and sends data to the adjacent nodes by a wireless transceiver module. Through the nodes of the sensor network, data are transmitted level by level until it is sent to the base station. Finally, data are transmitted to the host by the base station through the serial port to successfully monitor the location of the control.

The commonly used network transmission models include a star structure, a mesh structure, and a hybrid network. Each network structure exhibits its own advantages and defects. In fact, different network topologies should be selected according to different application requirements. Star topology is a single-hop system that supports point-to-point and point-to-multipoint communication. The structure requires low power consumption, but its transmission distance between node and base station is limited. The transmission distance of the frequency band is usually in meters. Star topology is suitable for the network of round equipment near distance. Mesh topology is a multi-hop system. By using multi-hop routing communication, all wireless sensor nodes perform identical roles, not only communicate directly with each other but also conducting data transmission and mutual transmission commands with the central node. Given that each sensor node possesses multiple paths to the center node or other nodes, the network exhibits strong robustness and reliability. The mesh topology system reaches farther transmission distance than the star-type structure and is suitable for large physical space-node dispersed systems. However, this system requires large power consumption.

**Figure 6:**
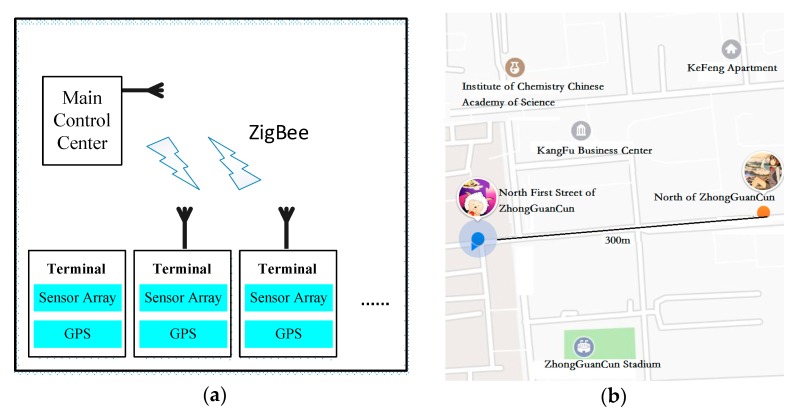
(**a**) Sketch map of the wireless communication module used in this study; (**b**) Position located by GPS in practical experiments.

Given the transmission distance requirement in this project, the system adopts mesh-network topology, and its network architecture is shown in Figure 6. Figure 6a is the sketch map of the established wireless communication module. In Figure 6b, the GPS positioning module (yellow point in right) is designed in the node, transmitting the location and detection data to the main control node (blue point in left) and meeting the required communication distance (300 m).

### 3.4. Actual Wireless Communication of the SAW Sensor Array

**Figure 7:**
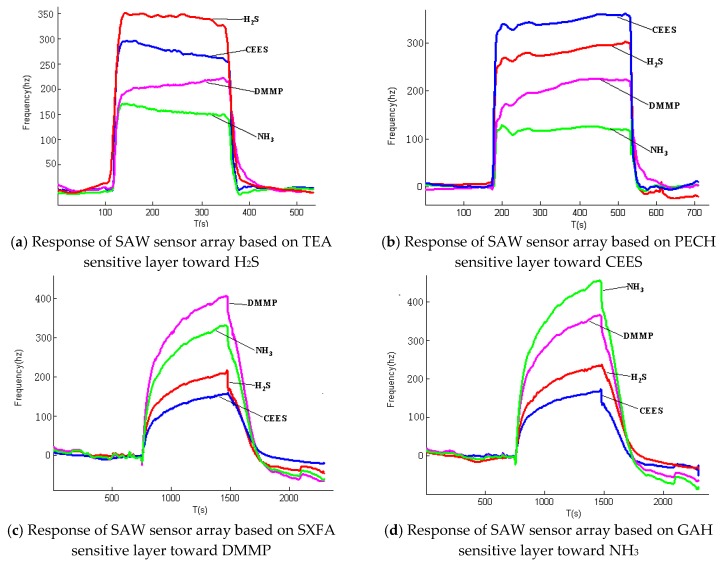
Detection results of the SAW sensor array to different gases.

Actual wireless communication experiments were conducted within 300 m outdoors. When H_2_S, CEES, DMMP, and NH_3_ passed through the SAW sensor array with the concentration of 10.76, 2.94, 2.96 and 21.53 ppm, respectively, response shifts are recorded by the SAW sensor array and transmited to the main control node (PC computer with Zigbee and Lora controllers), and the graphs formed by the detected data and testing position are visible on the screen of the PC computer in real time. The detected results are shown in Figure 7.

**Figure 8:**
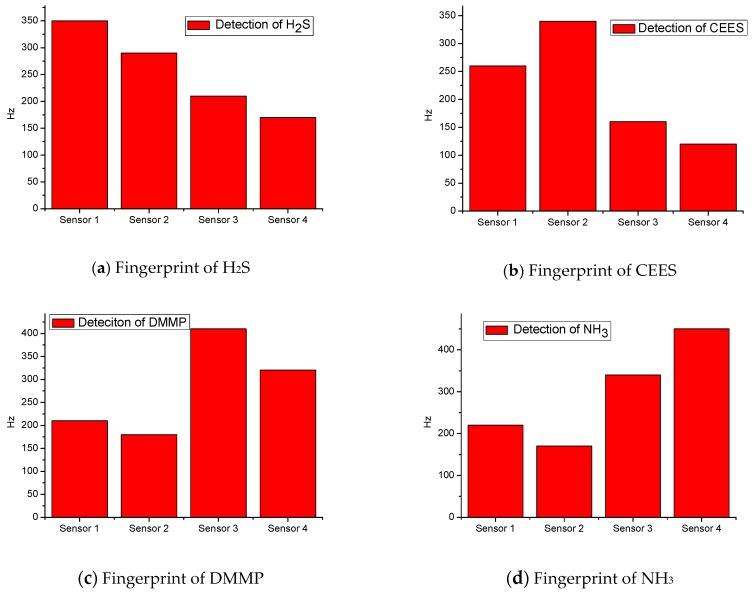
Fingerprints of the four kinds of gases detected by SAW sensor array.

Figure 7 indicates the whole processes when the four kinds of gases are detected using the developed SAW sensor array, and the variations are evident when different gases are detected. For example, when H_2_S gas passes through the sensor array, sensor 1 exhibits the largest signal response and the fastest response speed to H_2_S, whereas the other three sensors show relatively long response times and small response signals. This finding is attributed to the TEA coating of sensor 1, which is specific and sensitive to H_2_S; thus, the H_2_S molecules can be easily absorbed on the surface of TEA and rapidly reach equilibrium. Although the other three sensors can respond to H_2_S, the response signals and speeds are highly different; evidently, sensor 2 (coated with PECH to detect CEES) of the array shows the second largest signal response, followed by sensor 3 and sensor 4 with the third and fourth largest signal response, respectively. Therefore, the difference of the four sensors in detecting H_2_S is determined, and the fingerprint of the sensory array to detect H_2_S is obtained. Similar findings are observed in the other three sensors when NH_3_, CEES, and DMMP are detected, and the fingerprints are shown in Figure 8.

With the preparation of wireless communication of the SAW sensor array, H_2_S, CEES, DMMP, and NH_3_ can be detected, and the detected data can be transferred within 300 m. Meanwhile, the response signals and response time are highly different when different gases are detected by the SAW sensor array, and the corresponding fingerprints vary owing to film qualities. The detected gases can be identified by pattern recognition algorithm.

## 4. Conclusions

WSN is widely used in small-scale environmental monitoring. With the evolution of computer systems, monitoring combined with wireless communication networks is one of the best approaches to evaluate the change of environment in the future. As the best technology to provide context awareness, the detecting platform based on wireless communication include numerous monitor nodes to monitor the detected gases [29,32]. Considering the lack of an objective method for determining whether harmful gases leak into large-scale environment, we present a new wireless SAW-based chemical sensor array composed of TEA, PECH, SXFA, and GAH.

This approach in detecting H_2_S, CEES, DMMP, and NH_3_ by wireless communication technology is also demonstrated in our study. The method indicates numerous special features of the designed sensor array, including the design of an efficient WSN for monitoring, low power consumption, data availability, confidentiality, and easy remote configuration. This monitoring system presented in this paper can easily determine other parameters of interest, such as sensing activity, transmission from sensor nodes to base station, data storage, or visualization. Therefore, the developed approach may be extensively used in determining harmful gases for monitoring a given area.

The response of the developed SAW sensor array toward H_2_S, CEES, DMMP, and NH_3_, as well as repeatability, is characterized in gas experiments. With the wireless communication network module, H_2_S, CEES, DMMP, and NH_3_ are detected within 300 m under the environmentally safe concentrations of 1.08, 0.59, 0.10, and 5.02 ppm, respectively, the sensitivities of different sensors of the sensor array are 32.4, 14.9, 78.1 and 22.6 Hz/ppm, respectively, and the testing location is determined by the GPS module integrated into the sensor array. Although wireless communication is a considerable challenge in sensing and substantial work should be focused on this aspect, the current system that we developed demonstrated the feasibility of the WSN sensor array and its possibility in application. The future work will focus on enhancing the performance parameters of the device and the system, and the database should be established to improve recognition accuracy.
